# Early ART Results in Greater Immune Reconstitution Benefits in HIV-Infected Infants: Working with Data Missingness in a Longitudinal Dataset

**DOI:** 10.1371/journal.pone.0145320

**Published:** 2015-12-15

**Authors:** Livio Azzoni, Russell Barbour, Emmanouil Papasavvas, Deborah K. Glencross, Wendy S. Stevens, Mark F. Cotton, Avy Violari, Luis J. Montaner

**Affiliations:** 1 The Wistar Institute, Philadelphia, Pennsylvania, United States of America; 2 Biostatistics Department, Yale School of Public Health, New Haven, Connecticut, United States of America; 3 Department of Molecular Medicine and Hematology, University of the Witwatersrand and National Health Laboratory Service, Johannesburg, South Africa; 4 Children’s Infectious Diseases Clinical Research Unit, Department of Paediatrics and Child Health, Stellenbosch University, Cape Town, South Africa; 5 Perinatal HIV Research Unit, University of the Witwatersrand, Johannesburg, South Africa; University of Pittsburgh Center for Vaccine Research, UNITED STATES

## Abstract

**Background:**

Early initiation of anti-retroviral treatment (ART) decreases mortality as compared to deferred treatment, but whether it preserves immune cells from early loss or promotes their recovery remains undefined. Determination of complex immunological endpoints in infants is often marred by missing data due to missed visits and/or inadequate sampling. Specialized methods are required to address missingness and facilitate data analysis.

**Methods:**

We characterized the changes in cellular and humoral immune parameters over the first year of life in 66 HIV-infected infants (0–1 year of age) enrolled in the CHER study starting therapy within 12 weeks of birth (n = 42) or upon disease progression (n = 24). A convenience cohort of 23 uninfected infants aged 0–6 months born to mothers with HIV-1 infection was used as controls. Flow cytometry and ELISA were used to evaluate changes in natural killer (NK) cells, plasmacytoid dendritic cells (pDC), and CD4^+^ or CD8^+^ T-cell frequencies. Data missingness was assessed using Little's test. Complete datasets for analysis were created using Multiple Imputation (MI) or Bayesian modeling and multivariate analysis was conducted on the imputed datasets.

**Results:**

HIV-1-infected infants had greater frequency of CD4^+^ T cells with naïve phenotype, as well as higher serum IL-7 levels than HIV exposed/uninfected infants. The elevated data missingness was completely at random, allowing the use of both MI and Bayesian modeling. Both methods indicate that early ART initiation results in higher CD4^+^ T cell frequency, lower expression of CD95 in CD8^+^ T cell, and preservation of naïve T cell subsets. In contrast, innate immune effectors appeared to be similar independently of the timing of ART initiation.

**Conclusions:**

Early ART initiation in infants with perinatal HIV infection reduces immune activation and preserves an early expansion of naïve T-cells with undiminished innate cell numbers, giving greater immune reconstitution than achieved with deferred ART. Both statistical approaches concurred in this finding.

## Introduction

Perinatal HIV-1 infection results in progressive immunodeficiency and death in absence of early antiretroviral therapy (ART) [1]. Untreated HIV-infected children have high levels of CD8^+^, low levels of CD4^+^ T cells with memory (CD45RA^-^) phenotypes [2], and reduced levels of both naïve CD4^+^ and CD8^+^ T cells [2, 3]. Loss of naïve T cells in progressive pediatric infection has been attributed to both impairment of thymic function, as evidenced by decreased T-cell receptor excision circles (TREC) detection (reviewed in [4]) and, at least in part, to increased differentiation towards mature memory phenotypes [5].

Viremia in neonates coincides with early and sustained microbial translocation supporting the role of ongoing immune activation in early disease if left untreated [6, 7]. Ongoing HIV viremia and T-cell activation cause loss of peripheral naïve T cells, accompanied by homeostatic alterations, aimed at increasing thymic output. These include increased circulating IL-7 that can be sustained until late disease stages [8, 9]. However, the prognostic value of IL-7 in predicting immune recovery on treatment remains controversial [8–11]. It remains unknown whether early ART initiation in infants (as compared to older children or adults) may cause retention of IL-7 levels in conjunction with immune reconstitution. Regarding activation, while the expression of CD38 is also considered a maturation marker [12, 13], CD38 expression on CD8+ T cells has predominantly been identified as a measure of immune activation [14] that decreases in response to ART-mediated viral suppression [15].

Decreases in innate cell frequencies and function are associated with late stages of disease progression in pediatric infection as observed in adults [16, 17], including decreased pDC and reduced antibody dependent cytotoxicity (ADCC) capability to CD4^+^-infected targets [18]. How viremia and ART impact immune activation of innate and T-cell changes in perinatal infected infants, and how these children compare to age-matched exposed uninfected controls remains undetermined. We now address this question in infants from the “Children with HIV Early antiretroviral (CHER) trial [19]. This study demonstrated that administration of ART within the first 6–12 weeks of age results in higher survival than delaying therapy until infants are symptomatic [20]. As a result, early treatment is now recommended for all perinatally infected infants [20].

One of the issues encountered when studying infants is the high likelihood of missing data. This is particularly true in the case of heavily sampled infants and young children, where blood or tissue specimens are prioritized for safety assessments, leaving other assessments more likely to be triaged at the sample allocation stage. Longitudinal studies with repeated sampling are also likely to accumulate missingness due to skipped study visits, particularly in resource constrained settings where access to a central sampling location may present a difficulty. A number of statistical methods have been proposed for handling missing data. Some approaches have intrinsic problems: the last observation carried forward (LOCF) method may be appropriate for some “intent to treat” analyses, but not for evaluating variables that are anticipated to change over time. The Missing data Assumed to be Normal (MAN) method, which imputes the population mean for missing variables, is also inappropriate as it artificially reduces the standard error. Methods to overcome these limitations include mixed effect models and imputation (MI)-based general estimating equations (MI-GEE), with mixed performance ratings [21].

The Multiple Imputation (MI) method [22] imputes values for each missing cell in a data matrix, creating multiple "completed" data sets. In this process, the observed values remain the same, but the missing values are filled-in with different imputations to reflect uncertainty about the missing data. The major benefit of the MI method is that it does not change any relationships in the data otherwise, enabling inclusion of all the observed data in the partially missing rows. While using data imputation in predictive models has been considered unfavorably by some authors [23], it has been supported by others [24].

As noted by Weins and Moen [25], Bayesian simulations can accurately reconstruct highly incomplete biological datasets. Rubin classified data missingness in three categories [26]: a) missing completely at random (MCAR), where missingness is unassociated to any measured or unmeasured variable, b) missing at random (MAR), where the pattern of missingness of a variable is associated with the level of another measured independent variable, and c) missing not at random (MNAR), where missingness of a variable is associated with the level of the dependent variable. MCAR is considered the best possible type of missingness, because it does not introduce bias in the dataset. Although censoring the records containing missing information (complete case, listwise deletion) should not introduce bias [26, 27], MCAR, using robust methods to handle data missingness without censoring, maintains the power of the analysis [28].

Bayesian inference may be applied when the missingness is ignorable, i.e. either missing completely at random (MCAR) or missing at random (MAR) [29]. The recent release of robust software packages that simplify the simulation process and provide diagnostic capabilities for a robust approximation of missing data (e.g.: WinBugs [30] or the arm package [31] in R 3.1.2 (https://cran.r-project.org/bin/windows/base/old/3.1.2) has made Bayesian simulation methods more accessible.

Here we use MI and Bayesian modeling to analyze the impact of timing of ART initiation on innate and adaptive immune peripheral blood cell subsets in infants with perinatal HIV infection observed through their first year of life, using a dataset with high missingness.

## Material and Methods

### Patient population

HIV-1-infected and HIV-negative infants from HIV-infected mothers (control) were recruited at the Perinatal HIV Research Unit (PHRU), Chris Hani Baragwanath Hospital (Soweto, South Africa). All HIV infected infants were enrolled in the CHER trial [19] which randomized 6–12 week old HIV infected infants to deferred ART until the CD4^+^ T cell count dropped below 20% (ART-Def) or to start ART at time of enrollment (i.e. 6–12 week-old, ART-Early). The immune study described here was conducted in CHER participants and age-matched controls of up to 1 year of age. Data was collected once per semester (defined as a 6 month period in the year) in the following groups:

1. Group 1 (ART-Def) includes 28 infants who completed visit 1 (first semester) of which 14 infants completed visit 2 (second semester). Antiretroviral treatment with Zidovudine, Lamivudine, Lopinavir/Ritonavir) was initiated based on CD4^+^ T cell % or clinical criteria.
2. Group 2 (ART-Early) includes 42 infants who completed visit 1 (first semester) of which 29 infants completed visit 2 (second semester). Antiretroviral treatment with Zidovudine, Lamivudine, Lopinavir/Ritonavir) was initiated at enrollment.
3. Group 3, (HIV exposed uninfected [HEU] controls) is a convenience cohort of 23 uninfected infants aged 0–6 months, born to mothers with HIV-1 infection. HIV negativity was established at age 4–6 weeks using a single HIV DNA PCR test. In contrast to the HIV infected infants, the HEU controls had a single visit only.

Two consecutive blood samples were collected for the reported analyses, one during the first semester and one in the second semester. Due to the difficulty of obtaining sufficient blood volumes from infants, the blood-draw schedule was flexible within each 6-month period. A single sample was obtained from HIV exposed-uninfected infants in the control group, either in the first or second semester. All HIV-infected infants initiated ART by end of year 1.

#### Participant consent and ethics oversight

Written informed consent was obtained from parents or legal guardians of all participants. For infants enrolled in the CHER trial, a separate, written informed consent for the participation in this sub-study was obtained.

Ethics Committees of the University of the Witwatersrand, Stellenbosch University and the Wistar Institute Institutional Review Board approved consent forms, study protocols and relevant ethical issues.

### Flow Cytometry and serum IL-7 assessment

T lymphocyte and innate cell (DC, NK) subsets were evaluated using whole blood-based flow cytometry, using the following mouse monoclonal antibody combinations supplied in lyophilized 96-well plates (BD Biosciences, San José, CA): panel 1, T cells naïve/memory: CD45RA-FITC, CD27-PE, CD3-PerCP, CD4-APC; panel 2: T cell activation: CD7-FITC, CD95-PE, CD3-Per-CP, CD8-APC; panel 3: T cell activation: CD8-FITC, CD38-PE, CD3-PerCP, CD28-APC; panel 4: NK subpopulations: CD56FITC, CD16-PE, CD3-PerCP, CD161-APC; panel 5: pDC: Lin-1-FITC, CD123-PE, HLA-DR-PerCP, HLA-ABC-APC.

Flow cytometry was performed using a FACSCalibur Flow Cytometer using CellQuest software (BD Biosciences). Isotype-matched control antibodies were used as negative controls for gate positioning. Mean Fluorescence Intensity (MFI) of CD38 staining was assessed as described previously [32]. A total of 13 variables representing target subsets were analyzed.

IL-7 was measured on cryopreserved plasma using ELISA kits from R&D, Minneapolis, MN, following manufacturer’s directions, with a detection range of 0.25–16 pg/mL.

### Statistical analysis

**Fig 1:**
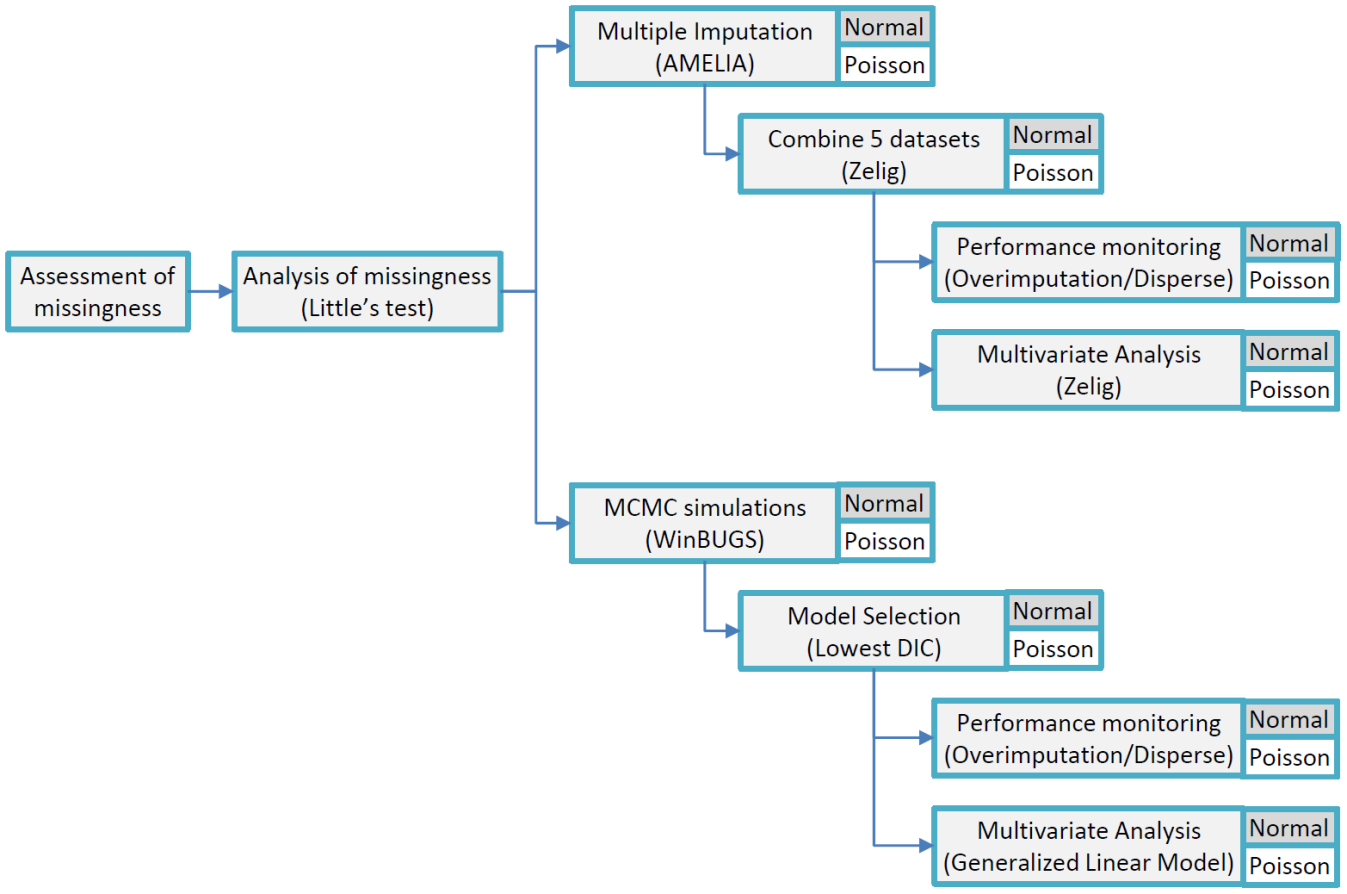
Statistical analysis summary. The tree represents the statistical analyses applied in the order in which they were performed. Distribution assumptions are indicated. Statistical packages are listed; where not specified, tests were conducted using R.

The overall statistical analysis approach is summarized in Fig 1.

#### Missingness analysis

**Fig 2:**
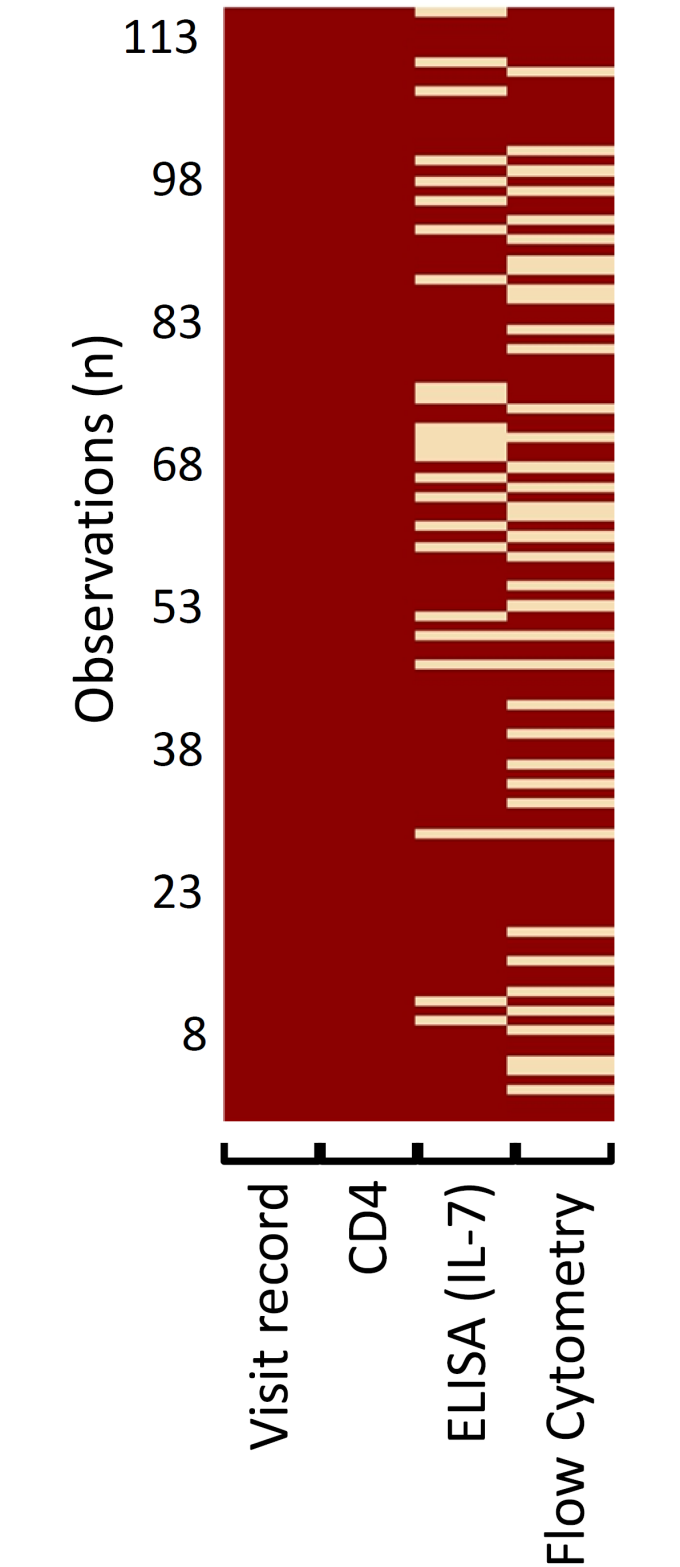
Missingness map. Data missingness across all observation for infants with data available for both visit 1 and 2 (visit record). All infants had CD4^+^ T cell % assessments at each recorded visit (CD4; missingness = 0%). IL-7 ELISA assessments were missing for 20% of the recorded visits; flow cytometry assessments were missing for a median of 31% (min 31%, max 34%) of the recorded visits.

In our longitudinal data set (HIV-infected infants), data for key variables incomplete in > 40% of the participants. A map of the observed missingness for the main variable categories is provided in Fig 2. We conducted two analyses to determine if the missingness was informative and therefore non-ignorable. Of particular concern was the possibility that missing data was due to poor health of the child, which could affect the treatment outcome. Our field staff reviewed chart data and confirmed that the corresponding study visits were not missed, but rather specimens were either not received at the laboratory, or were of insufficient volume/quality.

All subjects had a visit accession code and a CD4^+^ T cell % observation, which was obtained as first priority, but overall up to 40% of observations were missing from additional immunology variables (flow cytometry and/or ELISA data).

To assess whether the missingness was significantly related to either the dependent or independent variables (and therefore non-ignorable), we examined the structure of missing data by using Little’s Missing Completely at Random Test (MCAR) [33] using code developed for SAS software.

#### Multiple imputation (MI)

**Fig 3:**
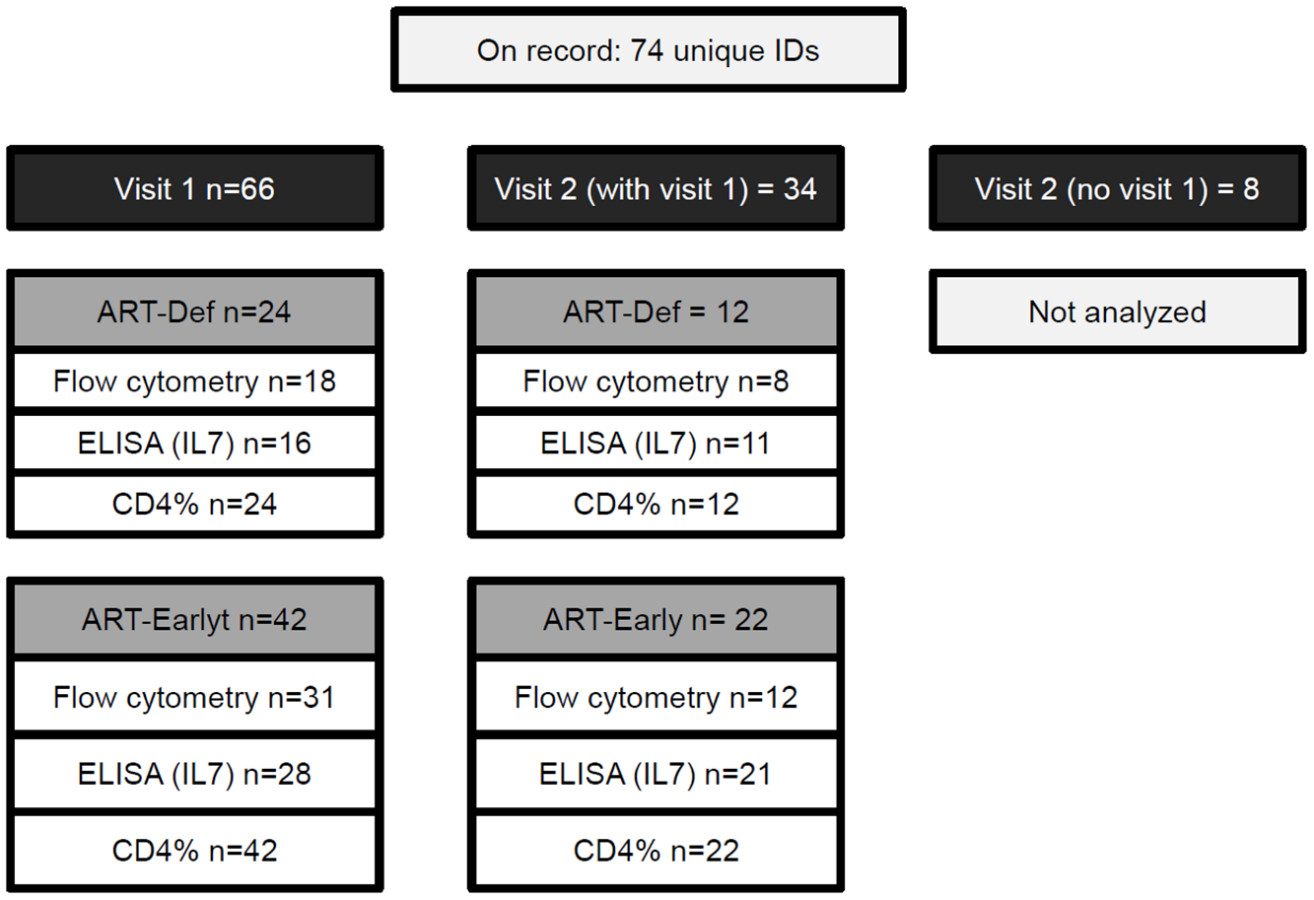
Data point availability. Subjects are grouped according to study visit (dark grey boxes; visit 1 = 1^st^ semester, visit 2 = 2^nd^ semester), and according to study group (light grey boxes; ART-Def = deferred treatment; ART-Early = early treatment). For each group, the number of tests available for analysis type is indicated (open boxes).

Software: we applied Multiple MI using the Amelia II package in R software [34]. We analyzed all records where the same child had both clinic visits documented (n = 34), but were missing flow cytometry and/or ELISA data (Fig 3).

We set the program at the recommended five imputations per dataset, which were then combined to a single imputed dataset. This was accomplished using the “mi” function in the Zelig add-on package in R [35, 36]. Zelig combined the five imputed dataset using Rubin’s rule [37] that accounts for both the ‘within’ and ‘between’ standard error of the imputed estimates before they are averaged.

Analysis assumptions: the Amelia II program assumes that the structure of the missingness is either MCAR or MAR [34].

Performance monitoring: the Amelia II package contains a number of algorithms to monitor performance of the Multiple Imputation process. Of the available metrics, we implemented the “overimputation” and “disperse” functions. Graphical representations that indicate the differences between observed (known) and imputed values were used to assess the performance. The results of this process for a representative variable (naïve CD27^+^/CD4^+^ T cells) are illustrated. (S1 Fig) We achieved normal Expectation—Maximization (EM) convergence. To assure EM convergence, we used the visual diagnostic “disperse” function from multiple over-dispersed starting values for output from Amelia.

Multivariate analysis: for multivariate analysis we combined the five imputed datasets using the Zelig [35] package version 4.1–3 in R (http://cran.r-project.org/web/packages/Zelig/index.html) which has a specific multiple imputation function “mi” to combine imputed data. The effect of arm assignment on the levels of all variables was assessed applying logistic regressions to the imputed data set using Zelig, using the AIC and “step “function in R for backward stepwise model selection.

#### Bayesian modeling

Software: to further explore the relationships suggested by analyses of the imputed dataset, we created Bayesian Simulations using two software packages. The initial simulations were performed using winBUGS [30] (version 1.4.3) with the BugsXLA interface [38, 39] to take advantage of its extensive diagnostic tools. The final analysis was performed with the recently released “arm” package in R [31] (version 1.6). In both packages the Bayesian analysis is based on Markov Chain Monte Carlo sampling, allowing us to implement an algorithm of 50,000 (winBUGS) or 100,000 (arm) simulations in the models presented here. In all of the simulations, the first 4000 initial MCMC samples were discarded (“burn-in”) under an assumption of convergence past this point [39].

Analysis assumptions: all priors were derived from observed data. We initially assumed a normal distribution for the independent effects and covariate regression coefficients as prior distributions. We excluded other prior distributions using the Deviance Information Criterion (DIC) in winBUGS that is reported in the BugsXLA output and as implemented in the “arm” package. We re-ran the simulations and models using a Poisson distribution in both software packages, which appeared closer to the observed distribution.

*Assumptions on distributions and data transformation*: The selection of the Poisson distribution is consistent with using proportional data in some instances [40]. The author suggests that in some instances a Poisson distribution might be an appropriate distribution for proportional data if not clustered at either bound of 0 or 1. We note that none of the proportional data of immunological factors presented in this study clustered at either 0 or 1, but were mostly ranged between 0.2 to 0.8.

Model selection: as suggested by Spiegelhalter et Al. [41], we chose the model with the lowest DIC value, which indicates that the model best predicts a replicate dataset which has the same structure as that currently observed.

Performance monitoring: to assess the model performance we monitored the Gelman and Rubin convergence statistics [42, 43] using winBUGS. This metric uses multiple simulated MCMC chains and then compares the variances within each chain and the variance between chains. The authors noted that a large deviation between these two variances indicates non-convergence. As illustrated in S2 Fig (representative variable CD27^+^/CD4^+^ T cells), our model resulted in good convergence, as diagnosed by the Gelman Rubin statistic approaching a value of 1.

Multivariate analysis: finally, to evaluate the effect of arm assignment and time on the variables at visit 2, we used the Bayesian estimated regression parameters and the estimates of the standard error and confidence limits to derive a multivariate Generalized Linear Model, with a significance level of *p* = 0.05.

As noted by Mason et Al. [44], the Full Bayesian Model that we have applied is a one-step procedure where imputation and analysis models are fitted simultaneously and the imputation model uses the joint distribution of all missing variables and applies the full posterior distribution of missing values in building a statistical model. In contrast MI is a two-step process with separate imputation and analysis models where the imputation model is based on a set of univariate conditional distributions.

## Results

### Infants with perinatal HIV-1 infection have higher IL-7, higher frequency of naïve CD4^+^ T cells and retained innate effectors

We sought to evaluate effects of perinatal HIV replication by evaluating changes in T-cell frequencies, immune activation and innate effectors. We first compared our cohort of 66 HIV-infected infants at visit 1 with a convenience cohort of 23 HEU infants. At the time of sampling, the ART-mediated suppression rate was 44% (29 of 66). As indicated in Table 1, after adjustment for multiple testing HIV-infected infants had a significantly lower % of total CD4^+^ T cells and higher % of activated (HLA-DR^+^) CD8^+^ T cells than HEU infants of comparable age, in keeping established observations in African children [14, 45]. An elevated expression (MFI) of CD38 was also observed on CD8^+^ T cells from the HIV-infected group, but the result was not significant after adjustment for multiple testing. HIV-infected infants also showed higher frequency of IL-7 and of CD4^+^ T cells with naïve phenotype (i.e.: CD45RA^+^/CD28^+^), suggesting a possible compensatory enhancement of thymic output activity as a result of HIV infection and/or CD4^+^ T cell depletion. NK and pDC frequencies were retained at control levels. Specifically, baseline levels of mature CD161^+^/56^+^/16^+^ and immature CD161^+^/56^-^/16^-^ NK cells, as well as pDC were similar in HIV infected and control infants (Table 1) suggesting that viremia in infants does not result in loss of innate cell subsets as observed in adults with acute or chronic infection [46–48] or infants with poorly controlled chronic infection [49].

**Table 1:** Baseline values in HIV-infected infants compared to a convenience cohort of HIV-exposed, uninfected (HEU) infants.

	HEU			HIV-infected^1^			T-test
Variable	mean	SD	n	mean	SD	n	p
Visit age	129.7	54.7	23	112.7	35.9	66	0.1756
Birth weight	2573	703	23	2942	455	66	0.0257
CD4^+^ (%)	42.5	6.8	23	36.0	8.5	66	0.0005 *
CD38^+^ (% of CD8^+^)	97.8	2.2	20	98.2	1.8	48	0.5214
HLA-DR^+^ (% of CD8^+^)	8.0	8.8	20	20.9	18.3	49	0.0002 *
CD95^+^ (% of CD8^+^)	84.3	19.4	20	77.1	25.5	49	0.2105
CD161^+^/56^+^/16^+^ (% of NK)	60.3	15.7	20	53.5	16.4	49	0.1136
CD161^+^/56^-^/16^-^ (% of NK)	3.2	2.3	20	4.5	4.9	49	0.118
pDC	0.2	0.3	20	0.3	0.1	46	0.1957
CD28^+^ naïve (% of CD4^+^)	67.4	10.2	20	75.9	8.9	49	0.0027 *
CD27^+^ naïve (% of CD4^+^)	74.8	11.3	20	82.4	9.0	49	0.011
CD28^+^ naïve (% of CD8^+^)	51.4	18.1	20	50.9	20.7	49	0.9182
CD27^+^ naïve (% of CD8^+^)	76.8	12.7	20	66.9	19.9	49	0.0168
Central Memory (% CD4^+^)	22.7	8.0	20	20.6	8.5	49	0.3226
Central Memory (% CD8^+^)	13.0	5.2	20	15.9	10.3	49	0.1366
CD38 MFI (in CD8^+^)	526.9	278.2	20	870.6	657.3	48	0.0035
IL7 (pg/ml)	2.4	1.9	21	5.4	4.3	50	0.0002 *

* Significant after Bonferroni adjustment for multiple testing.

^1^ Documented viral suppression (HIV VL < 400) = 44% (29 of 66).

### Addressing MCAR data missingness in follow-up visit by imputation and Bayesian model approaches

As illustrated in Fig 3, of the 66 infants completing a sub-study visit in the first semester (Visit 1, 24 in ART-Def, 42 in ART-Early), only 34 (12 and 22, respectively) had data for a study visit in the second semester. While CD4^+^ T cell % was assessed for all infants, assessments for flow cytometry (13 variables) and serum-based ELISA (one variable, IL-7) were not completed for all subjects. The distribution of the observed values for each variable assessed at visit 1 and 2 is summarized in Table 2. For a more complete breakdown of the observed values, please refer to S1a Table (all infants, by visit) and S1b Table (all infants by study arm and by visit).

**Table 2:** Observed values.

	Visit 1 (3–6 months)						Visit 2 (6–12 months)					
	ART-Def			ART-Early			ART-Def			ART-Early		
Variable	mean	SD	n	mean	SD	n	mean	SD	n	mean	SD	n
Visit age	102.6	33.9	24	118.4	36.2	42	263.0	57.2	12	265.2	71.5	22
Birth weight	2962.9	551.7	24	2930.2	396.3	42	2727.1	390.4	12	2895.5	405.9	22
CD4^+^ (%)	32.9	7.9	24	37.7	8.4	42	34.5	7.6	12	37.9	6.0	22
CD38^+^ (% of CD8^+^)	99.1	0.8	18	97.6	2.0	30	97.8	3.8	8	97.7	2.5	12
HLA-DR^+^ (% of CD8^+^)	38.0	17.6	18	10.9	8.8	31	22.9	19.7	7	21.9	16.7	12
CD95^+^ (% of CD8^+^)	93.4	7.7	18	67.7	27.5	31	87.0	11.5	7	73.4	22.3	12
CD161^+^/56^+^/16^+^ (% of NK)	55.4	16.5	18	52.4	16.5	31	62.4	7.7	8	60.7	16.9	12
CD161^+^/56^-^/16^-^ (% of NK)	3.1	3.4	18	5.4	5.4	31	2.8	1.9	8	4.4	5.8	12
pDC	0.3	0.1	17	0.3	0.2	29	0.4	0.7	8	0.4	0.4	11
CD28^+^ naïve (% of CD4^+^)	76.1	9.5	18	75.7	8.7	31	69.7	7.4	8	70.3	11.3	12
CD27^+^ naïve (% of CD4^+^)	80.5	8.5	18	83.6	9.3	31	78.4	10.4	8	80.7	5.7	12
CD28^+^ naïve (% of CD8^+^)	40.8	20.5	18	56.7	18.7	31	36.7	18.7	8	49.6	18.4	12
CD27^+^ naïve (% of CD8^+^)	53.7	18.1	18	74.5	16.8	31	54.5	16.2	8	67.8	14.2	12
Central Memory (% CD4^+^)	19.7	8.3	18	21.1	8.7	31	17.7	11.2	8	22.6	8.4	12
Central Memory (% CD8^+^)	22.9	12.5	18	11.8	5.7	31	20.9	4.0	8	16.9	6.1	12
CD38 MFI (on CD8^+^)	1402.0	790.9	18	551.8	230.5	30	658.6	390.8	8	830.6	355.9	12
IL7 (pg/ml)	4.1	3.7	16	5.4	4.4	28	4.0	3.6	11	4.9	3.4	21
Log_10_VL	5.9	0.8	14	3.1	0.6	22	5.0	1.6	4	3.0	1.0	16
VL > 400	-	-	14	-	-	15	-	-	3	-	-	4

To assess whether the missing data were likely to bias the analysis, we applied Little’s test [33] to determine if the missingness was completely at random (i.e.: unrelated to measured or unmeasured characteristics, MCAR [40]), at random (i.e.: associated with another variable’s value, MAR) or not at random (i.e.: associated with the value of the missing variable itself). The results of Little’s test were not significant for any variable used in the analysis, indicating the missingness is consistent with an MCAR pattern [50]. Based on this and additional confirmation by clinical sites, we established with reasonable certainty that the missing data were related to reasons unrelated to the clinical or immunological condition of the infants, but were rather attributable to missed visits, insufficient or inadequate blood draws or laboratory errors. These findings supported our conclusion that data missingness was MCAR, thus not informative and ignorable.

**Fig 4:**
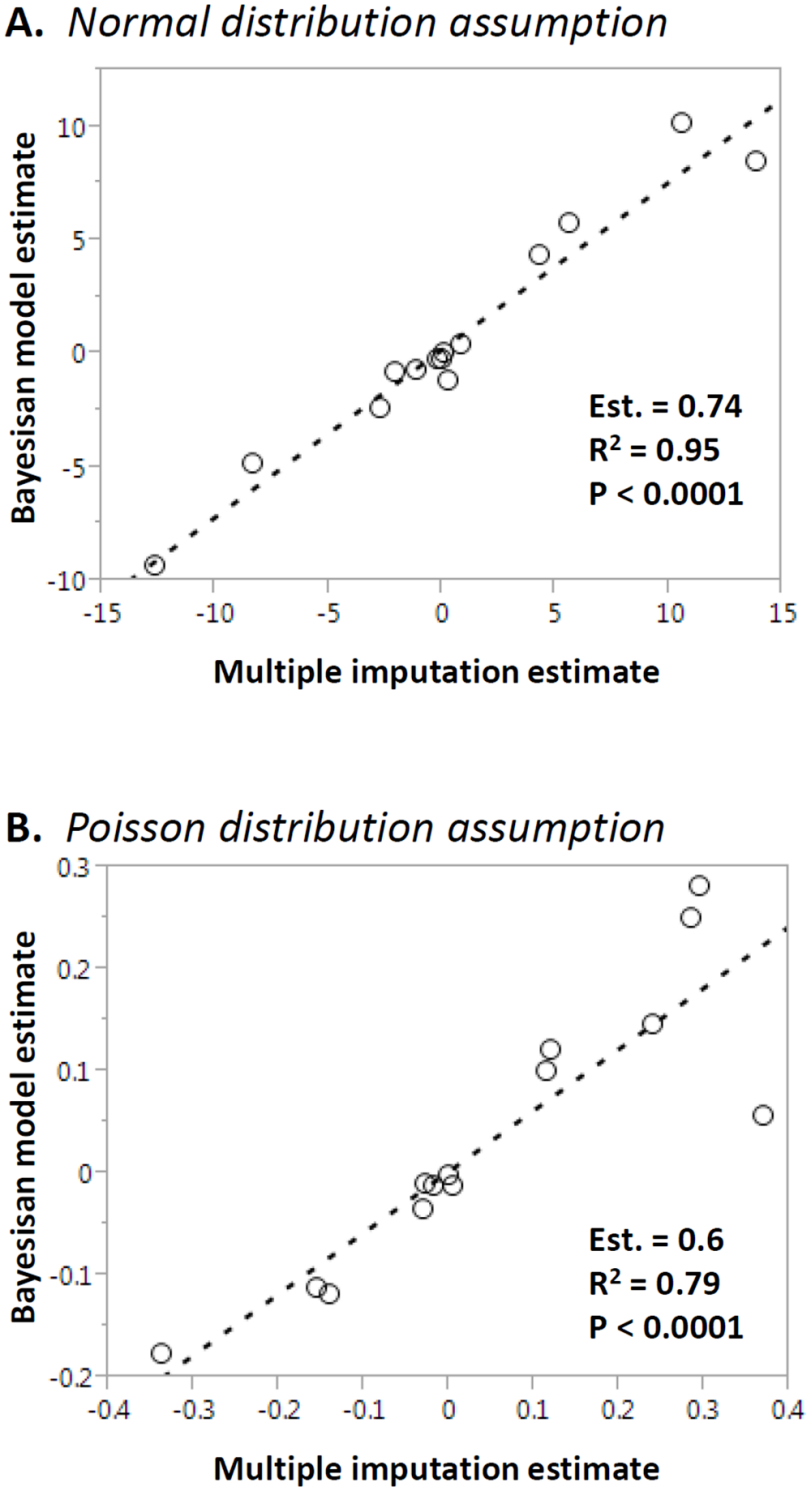
Method comparison. The means of the estimates for each variable obtained from Bayesian modeling (vertical axis) and Multiple Imputation (horizontal axis, transformed as indicated) were tested using Pearson’s linear regression. The slope estimate, R^2^ and p are indicated in the insets. Panel A: Normal distribution assumption; Panel B: Poisson distribution.

The observed data approximated a normal distribution for most variables. To assess the effect of distribution assumptions on the analysis, we also proceeded with a simple data transformation [log_e_ (var x 100)] allowing us to approximate a Poisson distribution. To compare the datasets created with MI and Bayesian modeling approaches, we first compared the means of the estimates obtained with the MI versus Bayesian modeling approaches for each variable. There was an overall good correlation between the estimates provided by both the Bayesian model approach and MI, under both a normal distribution assumption (Pearson P< 0.0001; R^2^ = 0.95; Fig 4A) and Poisson distribution assumption (Pearson P< 0,0001; R^2^ = 0.79; Fig 4B). The distributions of MI and Bayesian modeling estimates for each variable are provided in Tables 2 and 3, respectively. For a more comprehensive summary, please refer to S2 and S3 Tables.

**Table 3:** Multiple Imputation: univariate analysis with continuous variables imputed with treatment group as the independent variable.

	ART-Def		ART-Early—ART-Def Normal distribution			Lg(ART-Early)–Lg(ART-Def) Poisson distribution		
Response ^1^	Mean	St.Dev.	Estimate	SE	p	Estimate	SE	p
CD4^+^ (%)	38.27	1.28	4.337	2.204	0.049**	0.120	0.055	0.028**
CD38^+^ (% of CD8^+^)	97.52	0.47	0.023	0.837	0.979	0.001	0.033	0.972
HLA-DR^+^ (% of CD8^+^)	21.92	3.87	-8.373	7.732	0.283	-0.002	0.009	0.795
CD95^+^ (% of CD8^+^)	76.21	3.26	-12.651	6.239	0.044**	-0.154	0.046	0.003**
CD161^+^/56^+^/16^+^ (% of NK)	57.93	2.72	-1.085	5.430	0.842	-0.018	0.062	0.781
CD161^+^/56^-^/16^-^ (% of NK)	4.80	0.91	-0.172	1.658	0.917	-0.030	0.178	0.866
pDC	0.38	0.07	0.050	0.134	0.708	0.370	0.798	0.643
CD28^+^ naïve (% of CD4^+^)	71.34	1.71	-2.092	3.139	0.505	-0.028	0.040	0.480
CD27^+^ naïve (% of CD4^+^)	80.07	1.64	0.318	2.895	0.912	0.005	0.038	0.897
CD28^+^ naïve (% of CD8^+^)	46.11	3.42	10.620	7.524	0.165	0.286	0.052	0.000**
CD27^+^ naïve (% of CD8^+^)	65.76	3.16	13.889	5.809	0.017*	0.240	0.057	0.000**
Central Memory (% CD4^+^)	21.98	1.71	5.614	3.422	0.106	0.296	0.133	0.053*
Central Memory (% CD8^+^)	17.87	2.20	-2.732	4.176	0.513	-0.140	0.099	0.171
CD38 MFI (in CD8^+^)	808	1061	-88.02	242.318	0.719	-0.098	0.175	0.606
IL7 (pg/ml)	4.84	0.69	0.865	1.275	0.498	0.115	0.148	0.440

^1^ Predicted mean response with imputed data for early treatment group.

** Significant (p value < 0.05);

* Trend (p value < 0.1).

**Fig 5:**
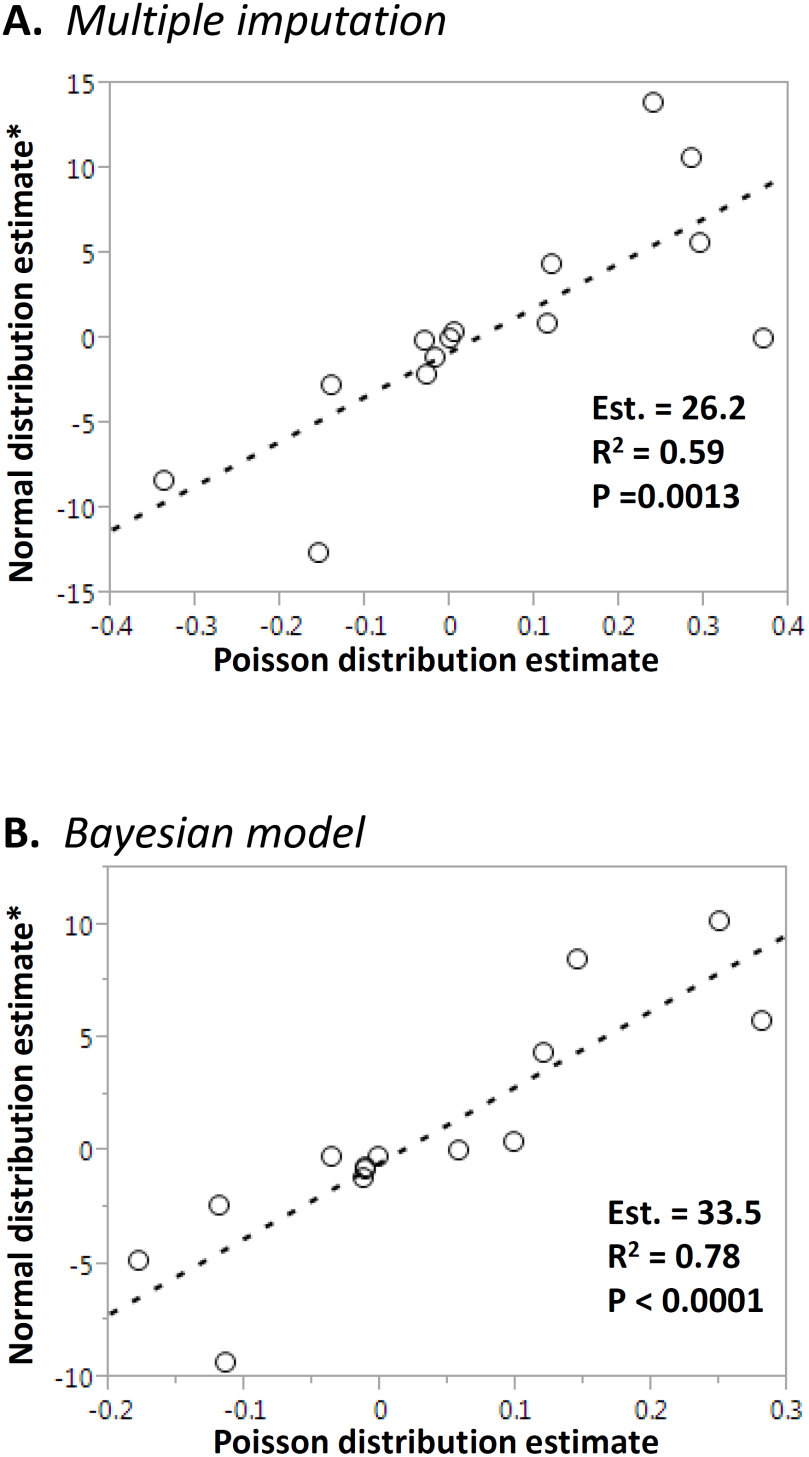
Effects of distribution assumptions on estimates. The means of the estimates for each variable obtained from Multiple Imputation (Panel A) or Bayesian modeling (Panel B) under a normal distribution assumption (assumption (vertical axis, transformed as indicated) or a Poisson distribution assumption (horizontal axis) were tested using Pearson’s linear regression. The slope estimate, R^2^ and p are indicated in the insets.

Within the same method, the effect of the distribution assumption was greater for the MI approach, as evidenced by the poorer linear fit between estimates across all variables (p = 0.0013; R^2^ = 0.59; Fig 5A). The correlation between estimates with normal and Poisson distribution assumption was slightly better for the Bayesian model (p< 0.0001; R^2^ = 0.78; Fig 5B).

Analysis of variables from visit 1 to visit 2 (detailed below) based on MI or Bayesian models showed that using a Poisson distribution assumption (with the required data transformations) yielded a larger number of significant comparisons (MI: 2 significant estimates for normal distribution vs. 5 with Poisson; Bayesian modeling: 1 significant estimates with Normal distribution vs. 7 with Poisson), supporting that an assessment of various distribution assumptions may be required to select the most appropriate method for any particular dataset.

### Early ART initiation results in greater CD4^+^ T-cell recovery and lower T-cell activation

We assessed the effects of early ART administration on CD4^+^ T cell % and T cell activation in both groups (Table 2). Median ART initiation was at 54 days (IQR 46; 61, n = 42) ART-Early and 220 days (IQR = 171; 341) for ART-Def. Of 21 infants in ART-Def group initiating ART after 180 day of age; 7 were not yet on ART by visit 2.

In our MI dataset (Table 3), the mean CD4^+^ T cell % of the early treatment group at the second semester visit (mean = 38.27% ± SD = 1.28) was significantly higher (difference estimate = 4.337 ± SE = 2.2, p = 0.049) than that of the ART-Def group under a normality assumption. This result was supported upon log_e_ transformation to approximate a Poisson distribution (difference estimate = 0.12 ± SE = 2.2, p = 0.028).

Similar results were also obtained with the Bayesian model (Table 4) with both normal (difference estimate = 4.337 ± SE = 2.152, p = 0.05) and Poisson (difference estimate = 0.12 ± SE = 0.054, p = 0.028) distributions.

**Table 4:** Bayesian model: univariate analysis with continuous variables Bayesian model without imputed data, with treatment group as the independent variable.

	ART-Def		ART-Early—ART-Def Normal distribution			Lg(ART-Early)–Lg(ART-Def) Poisson distribution		
Response ^1^	Mean	St.Dev.	Estimate	SE	p	Estimate	SE	p
CD4^+^ (%)	38.260	1.283	4.337	2.152	0.050*	0.12026	0.05477	0.028**
CD38^+^ (% of CD8^+^)	97.760	0.706	-0.242	1.124	0.831	-0.001702	0.041251	0.967
HLA-DR^+^ (% of CD8^+^)	22.100	5.555	-4.814	9.718	0.624	-0.17789	0.07987	0.026**
CD95^+^ (% of CD8^+^)	83.170	5.324	-9.350	7.749	0.238	-0.11352	0.04619	0.014**
CD161^+^/56^+^/16^+^ (% of NK)	57.350	4.283	-0.676	6.323	0.916	-0.01152	0.05361	0.830
CD161^+^/56^-^/16^-^ (% of NK)	4.536	1.341	-0.260	2.138	0.904	-0.03551	0.18789	0.850
pDC	0.276	0.053	-0.001	0.173	0.996	0.05716	0.86602	0.947
CD28^+^ naïve (% of CD4^+^)	70.630	2.602	-0.823	4.014	0.839	-0.01018	0.04842	0.834
CD27^+^ naïve (% of CD4^+^)	79.810	2.396	-1.209	3.818	0.754	-0.01328	0.04575	0.772
CD28^+^ naïve (% of CD8^+^)	45.880	5.455	10.145	8.166	0.225	0.24994	0.06548	0.000**
CD27^+^ naïve (% of CD8^+^)	64.560	4.617	8.486	7.356	0.259	0.14573	0.05384	0.007**
Central Memory (% CD4^+^)	22.990	2.535	5.728	3.940	0.158	0.28129	0.09367	0.003**
Central Memory (% CD8^+^)	18.830	3.422	-2.431	5.457	0.659	-0.11942	0.09047	0.187
CD38 MFI (in CD8^+^)	887	164	36.430	261.810	0.890	0.04207	0.01389	0.002**
IL7 (pg/ml)	4.698	0.692	0.443	1.151	0.702	0.09913	0.1612	0.539

^1^ Bayesian Model Predicting Mean Response for Early Treatment Group.

** Significant (p value < 0.05);

* Trend (p value < 0.1)

Viral replication is associated with elevated cellular activation, particularly in the CD8^+^ T cell compartment. Surprisingly, neither expression level nor frequency of CD38^+^/CD8^+^ T cells was significantly lower using the MI data whereas the Bayesian model approach (Poisson distribution, Table 4) did detect a change in CD38 MFI in CD8^+^ T cells as expected. This was further corroborated by a significant drop after ART in CD8^+^ T-cell frequencies expressing two other long-term activation or apoptosis-inducing proteins, HLA-DR or CD95, respectively. These results indicate that after addressing data missingness in visit 2 we could confirm the anticipated changes in infants receiving early ART where a higher CD4^+^ T cell immune recovery and lower expression of activation or pro-apoptotic molecules was significant.

### Early ART initiation results in the preservation of the Naïve T cell memory compartment and retention of T cell effector frequencies

Based on the higher CD4+ T cell recovery observed in ART-Early and to determine if early treatment has long-term effects on memory subset development or distribution, we assessed the frequency of naïve, central memory, intermediate memory and effector terminal CD4+ and CD8+ T cells. No significant difference was observed for CD4+ naïve T cell subsets in both MI and Bayesian sets. However, analysis of MI datasets (Table 3) indicated that at the second semester visit infants in ART-Early had a higher frequency of naïve (CD45RA+/CD27+) CD8+ T cells than infants in ART-Def (Normal distribution difference estimate = -13.889 ± SE = 5.809, p = 0.017. Poisson distribution difference estimate = 0.240 ± SE = 0.057, p< 0.001). This result was also supported by one of the Bayesian models (Poisson distribution difference estimate = 0.14573 ± SE = 0.05384, p = 0.007). When a Poisson distribution assumption was made, the same result was observed for the alternative naïve CD8+ phenotype (CD45RA+/CD28+) with both multiple imputation (Table 3. Difference estimate = 0.286 ± SE = 0.052, p<0.001) and Bayesian model (Table 4. Difference estimate = 0.25 ± SE = 0.06548, p<0.001).

Using a Poisson distribution assumption, CD45RA^-^/CD28^+^ CD4^+^ central memory T cells were higher in ART-Early at the second visit with both multiple imputation (Table 3. Difference estimate = 0.296 ± SE = 0.133, p = 0.053) and Bayesian models (Table 4. Difference estimate = 0.28129 ± SE = 0.09367, p = 0.003). Central memory CD8^+^ T cells were not significantly different between groups, independent of the method or distribution assumption. Interestingly, although baseline IL-7 levels were significantly higher in HIV-infected infants as compared to HEU controls (Table 1, p = 0.0002), and despite the increased frequency of naïve CD8^+^ T cells in ART-Early, serum levels of IL-7 at visit 2 were not significantly different between ART-Early and ART-Def, suggesting that its levels do not directly track the change in naïve T-cell frequencies observed in this cohort.

Finally, pre-ART levels of mature CD161+/56+/16+ and immature CD161+/56-/16- NK cells, as well as Plasmacytoid Dendritic cells remained stable, with change in both groups not significantly different after ART, independent of assessment method or the distribution assumptions (Tables 3 and 4).

## Discussion

We document for the first time that early ART initiation is associated with a greater recovery of CD4^+^ T-cells together with expanded CD4^+^ naïve T-cells. Elevated IL-7 during perinatal viremia was associated with greater expansion of CD8^+^ naïve T cells and retention of innate effector frequencies. Our findings were supported by statistical methods addressing elevated (> 40% for selected variables) MCAR data missingness. Addressing missing data was critical as the sample cohort provides the first large-scale assessment of immune benefits linked to the clinical benefits reported for the entire CHER cohort [51]. Comparison of multiple methods and distribution assumptions further largely confirmed the direction of the estimates, assisting interpretation of results and allowing inspection of concordance of the estimates values and direction as corroborative evidence.

In HIV-uninfected infants and young children, naïve T cells are the largest memory subset (reviewed in [52]). This subset is depleted in perinatally-infected infants with advanced disease. Here ART initiation results in a slow recovery of both CD8^+^ and CD4^+^ naïve T cells, with concomitant reduction of the effector memory T subsets and CD38+/HLA-DR+ activated T cells [53]. Early ART initiation preserves memory T and B compartment in pediatric cohorts [54, 55]. In infants from the parent CHER study, timing of ART initiation had no effect on quantitative humoral immune responses to a variety of vaccines [56]. However, qualitative responses to conjugated pneumococcal vaccine were significantly better in infants receiving early ART [57].

Our analysis shows a sustained elevation of IL-7 levels (no significant change over time), together with an increased frequency of CD4^+^ T cells with a naïve phenotype, in early treated HIV-infected infants. This suggests that early ART may promote the retention of an increase in thymic output during perinatal viremia. While the true level of thymic output in relation to the timing of ART in HIV-infected infants is still being evaluated in a CHER sub-study (e.g. via evaluation of TRECs), we interpret that the early increase in IL-7, supporting high thymic output, contributed to a rapid and higher recovery of the CD4^+^ T-cells, possibly contributing to the observed clinical benefits of early treatment (lower mortality and morbidity, [19]). We also document a rise in CD4^+^ T cell %, and a lower expression of activation and pro-apoptotic CD95 on CD8^+^ T cells upon viral suppression as expected. Interestingly, early treatment also resulted in greater proportion of CD8^+^ T cells with naïve phenotypes. These memory subsets are specifically affected during untreated HIV viremia; their sustained impairment in infants where ART is delayed may contribute to the worse clinical outcomes observed in ART-Def [19]. The observed increase in naïve CD8^+^ T cells associated with early treatment is also consistent with an increase in thymic output. While we cannot exclude that the lack of detection of significant differences in IL-7 levels after ART may be due to the limited sample size and lack of statistical power, it is interesting to speculate that retention of CD8^+^ naïve T cells, as observed with innate effectors, may contribute to greater T cell recovery in infants than adults receiving ART after acute infection. In this sense, retention of IL-7 levels after ART could be a positive prognostic factor in infants despite of viremia in contrast to HIV-infected adults [58, 59]. The interpretation that early ART in infants may result in greater benefits in immune reconstitution than adults is also supported by the fact that both NK cells and pDC subsets were retained irrespective of treatment strategy.

Analysis of longitudinal data after ART was dependent on addressing data missingness by Multiple Imputation and Bayesian Modeling. We elected not to censor incomplete records in order to retain power for detecting differences even though removing random missingness should not introduce analytical bias [28]. While a number of authors [22, 27, 60] have noted the superiority of both Multiple Imputation and Bayesian Modeling compared to complete case analysis or list-wise deletion, where missing data is simply eliminated, there remain substantial differences between the two methods. The multiple imputations approach allows us to address the question of likelihood of a dataset in the absence of missing data. The Bayesian modeling approach allows the creation of a large number of instances of datasets modeled on the observed data, thus increasing the power of the analysis. Of the two methods, using guidelines developed by these authors, both MI and Bayesian modeling are indicated when the dataset is small and the number of covariates with missingness is low. Both approaches have been successfully used in pediatric cohorts (examples in [61–64]), but not, to our knowledge, to support interpretation of prospective datasets with high missingness or to address immune variables in observational studies.

An important limitation of this work includes the small sample size and the relatively high missingness levels, suggesting a need to further validate our findings. Unlike the parent study, the work presented here is essentially observational, and thus is not adequately powered to draw conclusions on negative results. Therefore, any negative conclusion (e.g. lack of change in IL-7 levels over time etc.) should be interpreted with caution and confirmed in larger cohorts. Secondly, we did not study the functionality of immune subsets. Future work should determine if retained innate cell subsets undergo a functional change after ART as both IFN-α production by pDC [47] and IFN-γ production by NK cells [65] are impaired by viremia in HIV-infected adults independently of the relative cell frequency.

Another potential weakness of our approach is the influence of the data distribution assumptions on the analysis performance: in addition to the effects on the significance of the findings, the change in one variable (CD123+ pDC frequency) had a different direction depending on the distribution assumption (-0.001 for normal distribution vs. 0.058 for Poisson). Since this change was not significant under either assumption (p = 0.947), the significance of this divergence remains to be explored. Our data overall suggest that overall the Bayesian model may be less sensitive to distribution assumptions than the MI approach.

Lastly, our data does not address the long-term effects of ART, whether early or deferred, but rather focuses on early immune changes at 1 year of age.

In conclusion, despite its limitations, our analysis highlights the value of using statistical methods to work with high missingness dataset, particularly when extracting biological information from irreplaceable sample collections such as the CHER study analyzed here. Importantly, immune changes described after early ART ultimately highlight a limited window of opportunity following birth where ART can potentially preserve early immune compensatory mechanism during perinatal viremia to result in a more robust and clinically significant recovery of immune function during the first year of life.

## Supporting Information

S1 Fig Overimputation diagnostics (example): observed vs. imputed values of CD27^+^ naïve CD4^+^ T cells. Example of overimputation diagnostic (Amelia II package) showing the observed and imputed values of CD27^+^ CD4^+^ naïve T cells. As outlined by Honaker et al. [34], ninety percent confidence intervals are constructed that detail where an observed value would have been imputed had it been missing from the dataset, given the imputation model (i.e: how well would the model have predicted the known values had they been missing). The dots represent the mean imputation and the blue lines the confidence interval. Around ninety percent of these confidence intervals contain the y = x line, indicating that the true observed value falls within this range.
(TIF)

S2 Fig Convergence plots diagnostics (example). WinBUGS offers the Gelman-Rubin statistic for assessing convergence. In our example for values of CD4+/CD28+ Naïve T cells. This statistic assesses the variability within parallel chains (blue line) as compared to variability between parallel chains (green line). The model is judged to have converged if the ratio of between to within variability (red line) is close to 1. In our example convergence is indicated by the red line being close to 1 on the y-axis and by the blue and green lines being stable (horizontal) across the width of the plot for both runs (presented as separate panels). We used a conservative “burn in” where the first 4000 simulations were discarded. Parameter values that have been sampled at the beginning of the simulation are typically discarded so that the chain can converge to its stationary distribution. Large, conservative burn-in periods (as we applied) are generally preferable to shorter burn-in periods as noted by Merkle and Van Zandt [WinBUGS Tutorial Outline August 4, 2005 (http://www.stat.ubc.ca/lib/FCKuserfiles/WinBUGSforbeginners.pdf)]
(TIF)

S1 Table 1A. Observed values by visit. 1B. Observed values by visit and treatment arm.
(DOCX)

S2 Table Univariate analysis with continuous variables imputed (MI) and with treatment Group as the independent variable. (DOCX)

S3 Table Univariate analysis with continuous variables estimated using Bayesian Model and treatment Group as the independent variable. (DOCX)
