# Supplementary figures and images for: Early ART Results in Greater Immune Reconstitution Benefits in HIV-Infected Infants: Working with Data Missingness in a Longitudinal Dataset
Source: PLoS One. 2015 Dec 15;10(12):e0145320. doi: 10.1371/journal.pone.0145320 (PMC4699458; doi:10.1371/journal.pone.0145320)

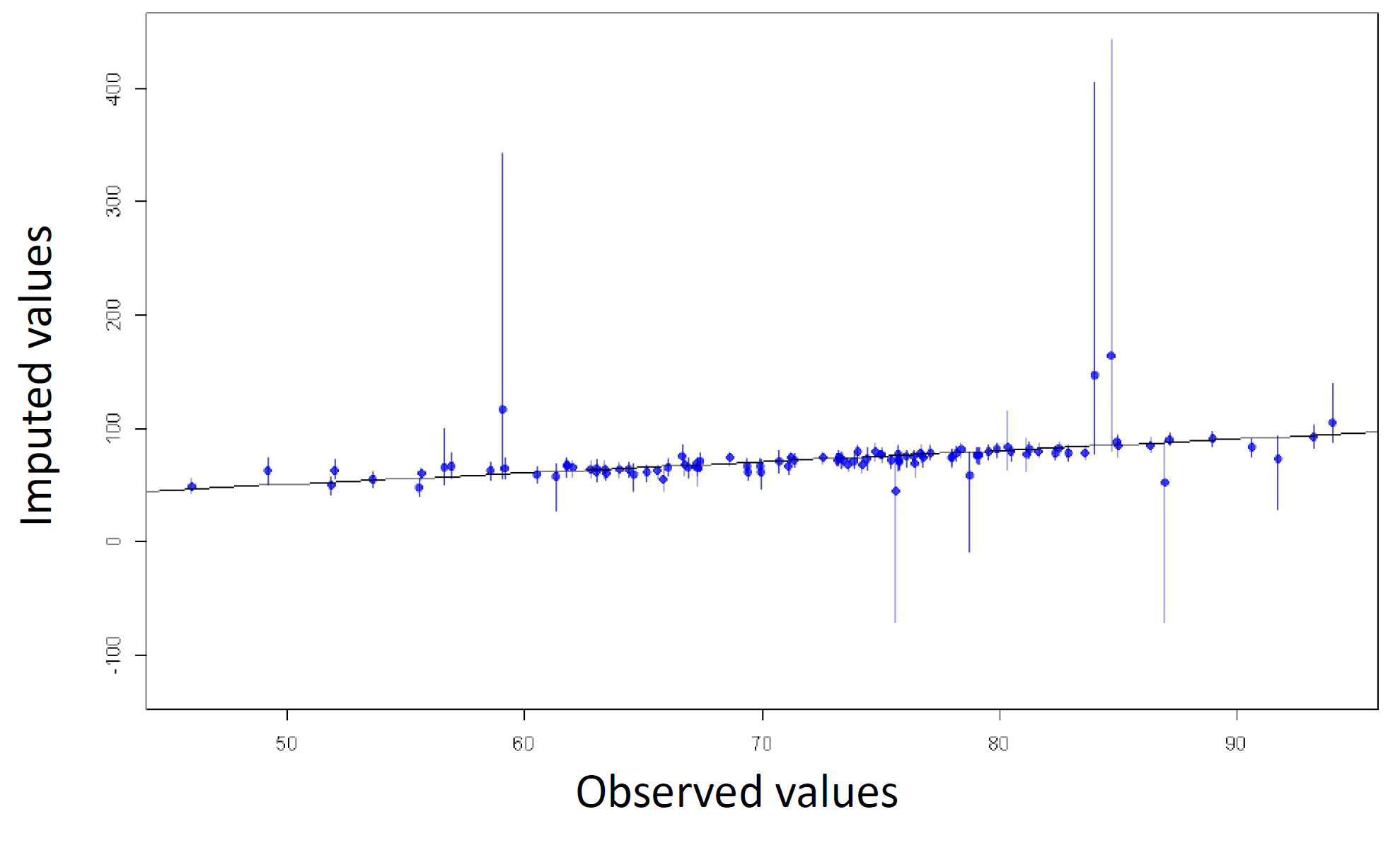

Supplement: S1 Fig — Example of overimputation diagnostic (Amelia II package) showing the observed and imputed values of CD27+ CD4+ naïve T cells. As outlined by Honaker et al. [34], ninety percent confidence intervals are constructed that detail where an observed value would have been imputed had it been missing from the dataset, given the imputation model (i.e: how well would the model have predicted the known values had they been missing). The dots represent the mean imputation and the blue lines the confidence interval. Around ninety percent of these confidence intervals contain the y = x line, indicating that the true observed value falls within this range. (TIF) [file pone.0145320.s001.tif]

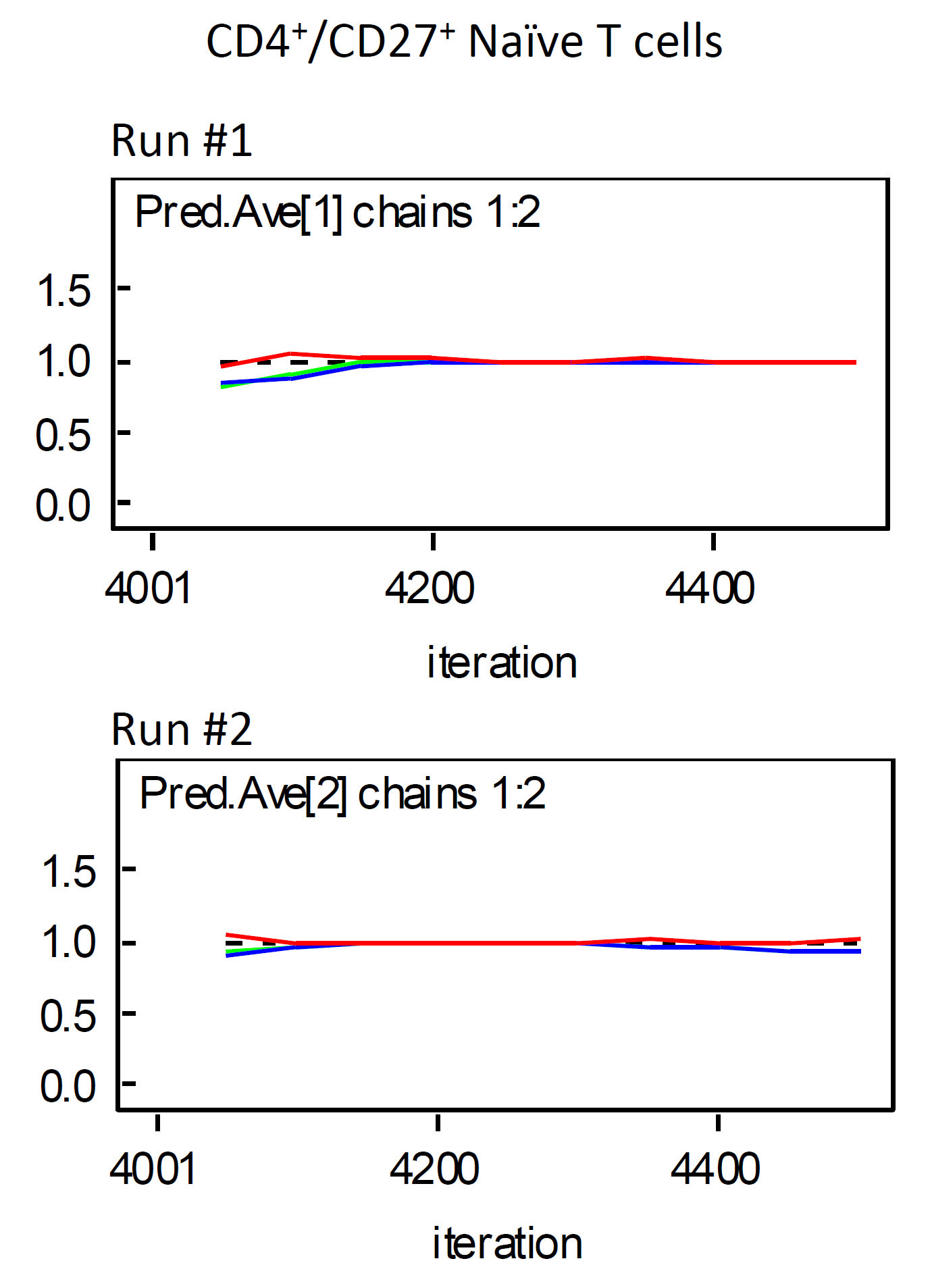

Supplement: S2 Fig — WinBUGS offers the Gelman-Rubin statistic for assessing convergence. In our example for values of CD4+/CD28+ Naïve T cells. This statistic assesses the variability within parallel chains (blue line) as compared to variability between parallel chains (green line). The model is judged to have converged if the ratio of between to within variability (red line) is close to 1. In our example convergence is indicated by the red line being close to 1 on the y-axis and by the blue and green lines being stable (horizontal) across the width of the plot for both runs (presented as separate panels). We used a conservative “burn in” where the first 4000 simulations were discarded. Parameter values that have been sampled at the beginning of the simulation are typically discarded so that the chain can converge to its stationary distribution. Large, conservative burn-in periods (as we applied) are generally preferable to shorter burn-in periods as noted by Merkle and Van Zandt [WinBUGS Tutorial Outline August 4, 2005 (http://www.stat.ubc.ca/lib/FCKuserfiles/WinBUGSforbeginners.pdf)] (TIF) [file pone.0145320.s002.tif]
