# Supplementary material for: Early ART Results in Greater Immune Reconstitution Benefits in HIV-Infected Infants: Working with Data Missingness in a Longitudinal Dataset
Source: PLoS One. 2015 Dec 15;10(12):e0145320. doi: 10.1371/journal.pone.0145320 (PMC4699458; doi:10.1371/journal.pone.0145320)
Supplement: S1 Table — 1A. Observed values by visit. 1B. Observed values by visit and treatment arm. (DOCX) [file pone.0145320.s003.docx]

**Supplemental Table 1**

Supplemental table 1A. Observed values by visit

|  | visit 1, all HIV-infected subjects | | | | | | visit 2, all HIV-infected subjects | | | | | |
| --- | --- | --- | --- | --- | --- | --- | --- | --- | --- | --- | --- | --- |
| Variable | mean | SD | Median | 2.5% | 97.5% | n | mean | SD | Median | 2.5% | 97.5% | n |
| Visit age | 112.7 | 35.9 | 107.5 | 57.0 | 172.4 | 66 | 264.4 | 66.0 | 255.0 | 182.8 | 358.1 | 34 |
| Birth weight | 2942.1 | 455.0 | 3000.0 | 2031.3 | 3731.9 | 66 | 2836.0 | 402.9 | 2877.5 | 2000.0 | 3617.5 | 34 |
| CD4^+^ (%) | 36.0 | 8.5 | 36.4 | 21.2 | 51.6 | 66 | 36.7 | 6.7 | 36.9 | 22.0 | 46.3 | 34 |
| CD38^+^ (% of CD8^+^) | 98.2 | 1.8 | 98.5 | 94.6 | 100.0 | 48 | 97.7 | 3.0 | 98.8 | 89.9 | 100.0 | 20 |
| HLA-DR^+^ (% of CD8^+^) | 20.9 | 18.3 | 15.6 | 1.6 | 53.5 | 49 | 22.2 | 17.3 | 15.9 | 2.2 | 51.0 | 19 |
| CD95^+^ (% of CD8^+^) | 77.1 | 25.5 | 88.0 | 19.5 | 99.9 | 49 | 78.4 | 19.8 | 84.1 | 40.4 | 98.2 | 19 |
| CD161^+^/56^+^/16^+^ (% of NK) | 53.5 | 16.4 | 57.0 | 23.5 | 76.5 | 49 | 61.4 | 13.7 | 59.8 | 41.0 | 80.6 | 20 |
| CD161^+^/56^-^/16^-^ (% of NK) | 4.5 | 4.9 | 2.5 | 0.2 | 16.4 | 49 | 3.8 | 4.6 | 2.3 | 0.4 | 15.7 | 20 |
| PDC | 0.3 | 0.1 | 0.3 | 0.1 | 0.6 | 46 | 0.4 | 0.5 | 0.2 | 0.0 | 1.6 | 19 |
| CD28^+^ naïve (% of CD4^+^) | 75.9 | 8.9 | 76.4 | 57.1 | 93.0 | 49 | 70.1 | 9.7 | 72.3 | 52.7 | 84.2 | 20 |
| CD27^+^ naïve (% of CD4^+^) | 82.4 | 9.0 | 83.2 | 58.8 | 96.5 | 49 | 79.8 | 7.7 | 80.9 | 65.1 | 91.9 | 20 |
| CD28^+^ naïve (% of CD8^+^) | 50.9 | 20.7 | 50.7 | 15.1 | 81.9 | 49 | 44.5 | 19.1 | 46.4 | 14.3 | 78.1 | 20 |
| CD27^+^ naïve (% of CD8^+^) | 66.9 | 19.9 | 69.9 | 30.4 | 94.9 | 49 | 62.5 | 16.1 | 60.6 | 33.6 | 89.6 | 20 |
| Central Memory (% CD4^+^) | 20.6 | 8.5 | 19.4 | 5.3 | 35.9 | 49 | 20.7 | 9.7 | 21.4 | 4.4 | 35.7 | 20 |
| Central Memory (% CD8^+^) | 15.9 | 10.3 | 13.5 | 3.8 | 40.6 | 49 | 18.5 | 5.6 | 18.3 | 8.1 | 26.5 | 20 |
| CD38 MFI (in CD8^+^) | 870.6 | 657.3 | 698.4 | 221.3 | 2279.7 | 48 | 761.8 | 370.3 | 684.6 | 325.6 | 1569.6 | 20 |
| IL7 (pg/ml) | 4.9 | 4.2 | 3.7 | 0.5 | 13.9 | 44 | 4.6 | 3.4 | 3.9 | 1.1 | 13.1 | 32 |
| VL | 896958 | 2053191 | 3540 | 393.8 | 7696250 | 36 | 129050 | 274117 | 399 | 399 | 750001 | 20 |
| Log_10_VL | 4.2 | 1.6 | 3.5 | 2.6 | 6.9 | 36 | 3.42 | 1.3 | 2.6 | 2.6 | 5.9 | 20 |
| VL > 400 | - | - | - | - | - | 29 | - | - | - | - | - | 7 |

Supplemental table 1B. Observed values by visit and treatment arm

|  |  | ART-Def | | | | | | ART-Early | | | | | |
| --- | --- | --- | --- | --- | --- | --- | --- | --- | --- | --- | --- | --- | --- |
|  | Variable | mean | SD | Median | 2.5% | 97.5% | n | mean | SD | Median | 2.5% | 97.5% | n |
| Visit 1 | Visit age | 102.6 | 33.9 | 98.5 | 56.6 | 157.4 | 24 | 118.4 | 36.2 | 113.5 | 60.1 | 173.0 | 42 |
|  | Birth weight | 2962.9 | 551.7 | 3045.0 | 2028.8 | 3961.4 | 24 | 2930.2 | 396.3 | 3000.0 | 2102.5 | 3596.3 | 42 |
|  | CD4^+^ (%) | 32.9 | 7.9 | 33.9 | 20.0 | 45.7 | 24 | 37.7 | 8.4 | 38.4 | 24.4 | 54.1 | 42 |
|  | CD38^+^ (% of CD8^+^) | 99.1 | 0.8 | 99.5 | 97.7 | 100.0 | 18 | 97.6 | 2.0 | 97.9 | 92.8 | 99.6 | 30 |
|  | HLA-DR^+^ (% of CD8^+^) | 38.0 | 17.6 | 39.6 | 8.2 | 69.5 | 18 | 10.9 | 8.8 | 10.1 | 1.4 | 32.6 | 31 |
|  | CD95^+^ (% of CD8^+^) | 93.4 | 7.7 | 97.3 | 77.6 | 100.0 | 18 | 67.7 | 27.5 | 72.4 | 17.4 | 99.4 | 31 |
|  | CD161^+^/56^+^/16^+^ (% of NK) | 55.4 | 16.5 | 55.9 | 25.0 | 77.9 | 18 | 52.4 | 16.5 | 58.4 | 25.1 | 74.3 | 31 |
|  | CD161^+^/56^-^/16^-^ (% of NK) | 3.1 | 3.4 | 1.8 | 0.1 | 11.3 | 18 | 5.4 | 5.4 | 3.5 | 0.5 | 17.6 | 31 |
|  | PDC | 0.3 | 0.1 | 0.3 | 0.1 | 0.5 | 17 | 0.3 | 0.2 | 0.3 | 0.1 | 0.6 | 29 |
|  | CD28^+^ naïve (% of CD4^+^) | 76.1 | 9.5 | 76.5 | 58.7 | 93.1 | 18 | 75.7 | 8.7 | 75.8 | 60.7 | 90.1 | 31 |
|  | CD27^+^ naïve (% of CD4^+^) | 80.5 | 8.5 | 80.4 | 64.0 | 94.2 | 18 | 83.6 | 9.3 | 85.4 | 57.6 | 95.8 | 31 |
|  | CD28^+^ naïve (% of CD8^+^) | 40.8 | 20.5 | 44.1 | 13.8 | 69.8 | 18 | 56.7 | 18.7 | 54.7 | 24.4 | 84.2 | 31 |
|  | CD27^+^ naïve (% of CD8^+^) | 53.7 | 18.1 | 50.1 | 25.8 | 84.2 | 18 | 74.5 | 16.8 | 77.7 | 42.8 | 95.5 | 31 |
|  | Central Memory (% CD4^+^) | 19.7 | 8.3 | 20.7 | 4.6 | 32.4 | 18 | 21.1 | 8.7 | 19.1 | 8.7 | 38.6 | 31 |
|  | Central Memory (% CD8^+^) | 22.9 | 12.5 | 20.6 | 4.5 | 47.0 | 18 | 11.8 | 5.7 | 9.4 | 4.1 | 23.9 | 31 |
|  | CD38 MFI (in CD8^+^) | 1402.0 | 790.9 | 1445.1 | 443.3 | 3009.2 | 18 | 551.8 | 230.5 | 461.0 | 195.0 | 962.8 | 30 |
|  | IL7 (pg/ml) | 4.1 | 3.7 | 2.2 | 1.0 | 12.3 | 16 | 5.4 | 4.4 | 5.0 | 0.4 | 14.6 | 28 |
|  | VL | 2292407 | 2813574 | 990000 | 28472 | 8559750 | 14 | 8945 | 34446 | 973 | 379 | 80884 | 22 |
|  | Log_10_VL | 5.9 | 0.8 | 6.0 | 4.4 | 6.9 | 14 | 3.1 | 0.6 | 3.0 | 2.6 | 4.5 | 22 |
|  | VL > 400 |  |  |  |  |  | 14 |  |  |  |  |  | 15 |

|  |  | ART-Def | | | | | | ART-Early | | | | | |
| --- | --- | --- | --- | --- | --- | --- | --- | --- | --- | --- | --- | --- | --- |
|  | Variable | mean | SD | Median | 2.5% | 97.5% | n | mean | SD | Median | 2.5% | 97.5% | n |
| Visit 2 | Visit age | 263.0 | 57.2 | 259.0 | 183.1 | 349.5 | 12 | 265.2 | 71.5 | 219.0 | 184.6 | 359.9 | 22 |
|  | Birth weight | 2727.1 | 390.4 | 2775.0 | 2110.0 | 3272.5 | 12 | 2895.5 | 405.9 | 2900.0 | 2052.5 | 3647.5 | 22 |
|  | CD4^+^ (%) | 34.5 | 7.6 | 35.2 | 20.6 | 44.1 | 12 | 37.9 | 6.0 | 37.5 | 26.6 | 47.5 | 22 |
|  | CD38^+^ (% of CD8^+^) | 97.8 | 3.8 | 99.4 | 90.2 | 100.0 | 8 | 97.7 | 2.5 | 98.7 | 92.5 | 99.9 | 12 |
|  | HLA-DR^+^ (% of CD8^+^) | 22.9 | 19.7 | 15.9 | 5.0 | 50.9 | 7 | 21.9 | 16.7 | 18.5 | 2.0 | 48.1 | 12 |
|  | CD95^+^ (% of CD8^+^) | 87.0 | 11.5 | 88.9 | 69.7 | 98.4 | 7 | 73.4 | 22.3 | 82.4 | 40.2 | 94.8 | 12 |
|  | CD161^+^/56^+^/16^+^ (% of NK) | 62.4 | 7.7 | 59.8 | 53.8 | 75.1 | 8 | 60.7 | 16.9 | 61.5 | 40.8 | 81.1 | 12 |
|  | CD161^+^/56^-^/16^-^ (% of NK) | 2.8 | 1.9 | 2.3 | 0.7 | 4.9 | 8 | 4.4 | 5.8 | 2.3 | 0.4 | 17.8 | 12 |
|  | PDC | 0.4 | 0.7 | 0.2 | 0.0 | 1.7 | 8 | 0.4 | 0.4 | 0.3 | 0.1 | 1.1 | 11 |
|  | CD28^+^ naïve (% of CD4^+^) | 69.7 | 7.4 | 72.3 | 57.7 | 78.1 | 8 | 70.3 | 11.3 | 72.6 | 51.2 | 84.4 | 12 |
|  | CD27^+^ naïve (% of CD4^+^) | 78.4 | 10.4 | 79.0 | 63.3 | 93.3 | 8 | 80.7 | 5.7 | 82.1 | 69.8 | 88.5 | 12 |
|  | CD28^+^ naïve (% of CD8^+^) | 36.7 | 18.7 | 33.9 | 12.9 | 63.4 | 8 | 49.6 | 18.4 | 50.4 | 21.5 | 80.6 | 12 |
|  | CD27^+^ naïve (% of CD8^+^) | 54.5 | 16.2 | 56.3 | 31.6 | 72.3 | 8 | 67.8 | 14.2 | 62.0 | 52.9 | 89.9 | 12 |
|  | Central Memory (% CD4^+^) | 17.7 | 11.2 | 20.1 | 3.3 | 31.9 | 8 | 22.6 | 8.4 | 21.4 | 12.1 | 36.8 | 12 |
|  | Central Memory (% CD8^+^) | 20.9 | 4.0 | 20.3 | 16.9 | 26.8 | 8 | 16.9 | 6.1 | 17.4 | 8.0 | 25.1 | 12 |
|  | CD38 MFI (in CD8^+^) | 658.6 | 390.8 | 518.4 | 279.9 | 1304.4 | 8 | 830.6 | 355.9 | 726.3 | 535.8 | 1623.0 | 12 |
|  | IL7 (pg/ml) | 4.0 | 3.6 | 3.1 | 1.1 | 11.6 | 11 | 4.9 | 3.4 | 4.1 | 1.1 | 12.9 | 21 |
|  | VL | 445600 | 366482 | 516000 | 22119 | 749400 | 4 | 49912 | 186855 | 399 | 399 | 480113 | 16 |
|  | Log_10_VL | 5.0 | 1.6 | 5.7 | 2.8 | 5.9 | 4 | 3.0 | 1.0 | 2.6 | 2.6 | 5.4 | 16 |
|  | VL > 400 |  |  |  |  |  | 3 |  |  |  |  |  | 4 |
