# Supplementary material for: Early ART Results in Greater Immune Reconstitution Benefits in HIV-Infected Infants: Working with Data Missingness in a Longitudinal Dataset
Source: PLoS One. 2015 Dec 15;10(12):e0145320. doi: 10.1371/journal.pone.0145320 (PMC4699458; doi:10.1371/journal.pone.0145320)
Supplement: S2 Table — (DOCX) [file pone.0145320.s004.docx]

Supplemental table 2. Univariate analysis with continuous variables imputed (MI) and with treatment Group as the independent variable

|  | ART-Def | | | | | ART-Early – ART-Def  Normal distribution | | | | Lg(ART-Early) – Lg(ART-Def)  Poisson distribution | | | |
| --- | --- | --- | --- | --- | --- | --- | --- | --- | --- | --- | --- | --- | --- |
| Response ^1^ | Mean | St.Dev. | 2.5% | Median | 97.5% | Estimate | SE | t | p | Estimate | SE | t | p |
| CD4^+^ (%) | 38.27 | 1.28 | 35.96 | 38.26 | 40.77 | 4.337 | 2.204 | 1.968 | 0.049 | 0.120 | 0.055 | 2.196 | 0.028 |
| CD38^+^ (% of CD8^+^) | 97.52 | 0.47 | 96.61 | 97.53 | 98.42 | 0.023 | 0.837 | 0.027 | 0.979 | 0.001 | 0.033 | 0.036 | 0.972 |
| HLA-DR^+^ (% of CD8^+^) | 21.92 | 3.87 | 14.64 | 21.82 | 29.55 | -8.373 | 7.732 | -1.083 | 0.283 | -0.002 | 0.009 | -0.260 | 0.795 |
| CD95^+^ (% of CD8^+^) | 76.21 | 3.26 | 70.14 | 76.23 | 82.54 | -12.651 | 6.239 | -2.028 | 0.044 | -0.154 | 0.046 | -3.336 | 0.003 |
| CD161^+^/56^+^/16^+^ (% of NK) | 57.93 | 2.72 | 52.87 | 57.85 | 62.98 | -1.085 | 5.430 | -0.200 | 0.842 | -0.018 | 0.062 | -0.284 | 0.781 |
| CD161^+^/56^-^/16^-^ (% of NK) | 4.80 | 0.91 | 3.08 | 4.83 | 6.51 | -0.172 | 1.658 | -0.104 | 0.917 | -0.030 | 0.178 | -0.170 | 0.866 |
| PDC | 0.38 | 0.07 | 0.25 | 0.38 | 0.51 | 0.050 | 0.134 | 0.376 | 0.708 | 0.370 | 0.798 | 0.464 | 0.643 |
| CD28^+^ naïve (% of CD4^+^) | 71.34 | 1.71 | 68.04 | 71.32 | 74.48 | -2.092 | 3.139 | -0.666 | 0.505 | -0.028 | 0.040 | -0.708 | 0.480 |
| CD27^+^ naïve (% of CD4^+^) | 80.07 | 1.64 | 77.00 | 80.11 | 82.96 | 0.318 | 2.895 | 0.110 | 0.912 | 0.005 | 0.038 | 0.130 | 0.897 |
| CD28^+^ naïve (% of CD8^+^) | 46.11 | 3.42 | 39.72 | 46.10 | 52.81 | 10.620 | 7.524 | 1.411 | 0.165 | 0.286 | 0.052 | 5.469 | 0.000 |
| CD27^+^ naïve (% of CD8^+^) | 65.76 | 3.16 | 59.68 | 65.73 | 71.41 | 13.889 | 5.809 | 2.391 | 0.017 | 0.240 | 0.057 | 4.239 | 0.000 |
| Central Memory (% CD4^+^) | 21.98 | 1.71 | 18.53 | 22.03 | 25.18 | 5.614 | 3.422 | 1.641 | 0.106 | 0.296 | 0.133 | 2.221 | 0.053 |
| Central Memory (% CD8^+^) | 17.87 | 2.20 | 13.54 | 17.83 | 22.22 | -2.732 | 4.176 | -0.654 | 0.513 | -0.140 | 0.099 | -1.418 | 0.171 |
| CD38 MFI (in CD8^+^) | 808 | 1061 | 614 | 807 | 1019 | -88.02 | 242.318 | -0.363 | 0.719 | -0.098 | 0.175 | -0.559 | 0.606 |
| IL7 (pg/ml) | 4.84 | 0.69 | 3.51 | 4.85 | 6.12 | 0.865 | 1.275 | 0.678 | 0.498 | 0.115 | 0.148 | 0.773 | 0.440 |

*^1^ Predicted Mean Response with Imputed Data for Early Treatment Group*
