# Supplementary material for: Early ART Results in Greater Immune Reconstitution Benefits in HIV-Infected Infants: Working with Data Missingness in a Longitudinal Dataset
Source: PLoS One. 2015 Dec 15;10(12):e0145320. doi: 10.1371/journal.pone.0145320 (PMC4699458; doi:10.1371/journal.pone.0145320)
Supplement: S3 Table — (DOCX) [file pone.0145320.s005.docx]

Supplemental table 3. Univariate analysis with continuous variables estimated using Bayesian Model and treatment Group as the independent variable.

|  | ART-Def | | | | | ART-Early – ART-Def  Normal distribution | | | | Lg(ART-Early) – Lg(ART-Def)  Poisson distribution | | | |
| --- | --- | --- | --- | --- | --- | --- | --- | --- | --- | --- | --- | --- | --- |
| Response ^1^ | Mean | St.Dev. | 2.5% | Median | 97.5% | Estimate | SE | Z | p | Estimate | SE | Z | p |
| CD4^+^ (%) | 38.260 | 1.283 | 35.740 | 38.270 | 40.780 | 4.337 | 2.152 | 2.016 | 0.050 | 0.12026 | 0.05477 | 2.196 | 0.028 |
| CD38^+^ (% of CD8^+^) | 97.760 | 0.706 | 96.360 | 97.770 | 99.150 | -0.242 | 1.124 | -0.216 | 0.831 | -0.001702 | 0.041251 | -0.041 | 0.967 |
| HLA-DR^+^ (% of CD8^+^) | 22.100 | 5.555 | 11.620 | 21.940 | 33.460 | -4.814 | 9.718 | -0.495 | 0.624 | -0.17789 | 0.07987 | -2.227 | 0.026 |
| CD95^+^ (% of CD8^+^) | 83.170 | 5.324 | 72.140 | 83.480 | 92.630 | -9.350 | 7.749 | -1.207 | 0.238 | -0.11352 | 0.04619 | -2.458 | 0.014 |
| CD161^+^/56^+^/16^+^ (% of NK) | 57.350 | 4.283 | 48.900 | 57.340 | 65.760 | -0.676 | 6.323 | -0.107 | 0.916 | -0.01152 | 0.05361 | -0.215 | 0.830 |
| CD161^+^/56^-^/16^-^ (% of NK) | 4.536 | 1.341 | 1.866 | 4.543 | 7.166 | -0.260 | 2.138 | -0.121 | 0.904 | -0.03551 | 0.18789 | -0.189 | 0.850 |
| PDC | 0.276 | 0.053 | 0.177 | 0.157 | 0.387 | -0.001 | 0.173 | -0.005 | 0.996 | 0.05716 | 0.86602 | 0.066 | 0.947 |
| CD28^+^ naïve (% of CD4^+^) | 70.630 | 2.602 | 65.510 | 70.640 | 75.760 | -0.823 | 4.014 | -0.205 | 0.839 | -0.01018 | 0.04842 | -0.21 | 0.834 |
| CD27^+^ naïve (% of CD4^+^) | 79.810 | 2.396 | 74.980 | 79.880 | 84.370 | -1.209 | 3.818 | -0.317 | 0.754 | -0.01328 | 0.04575 | -0.29 | 0.772 |
| CD28^+^ naïve (% of CD8^+^) | 45.880 | 5.455 | 35.090 | 45.910 | 56.570 | 10.145 | 8.166 | 1.242 | 0.225 | 0.24994 | 0.06548 | 3.817 | 0.000 |
| CD27^+^ naïve (% of CD8^+^) | 64.560 | 4.617 | 55.460 | 64.570 | 73.700 | 8.486 | 7.356 | 1.154 | 0.259 | 0.14573 | 0.05384 | 2.707 | 0.007 |
| Central Memory (% CD4^+^) | 22.990 | 2.535 | 18.000 | 22.980 | 28.020 | 5.728 | 3.940 | 1.454 | 0.158 | 0.28129 | 0.09367 | 3.003 | 0.003 |
| Central Memory (% CD8^+^) | 18.830 | 3.422 | 12.010 | 18.840 | 25.540 | -2.431 | 5.457 | -0.446 | 0.659 | -0.11942 | 0.09047 | -1.32 | 0.187 |
| CD38 MFI (in CD8^+^) | 887 | 164 | 560 | 888 | 1209 | 36.430 | 261.810 | 0.139 | 0.890 | 0.04207 | 0.01389 | 3.029 | 0.002 |
| IL7 (pg/ml) | 4.698 | 0.692 | 3.324 | 4.696 | 6.062 | 0.443 | 1.151 | 0.385 | 0.702 | 0.09913 | 0.1612 | 0.615 | 0.539 |

*^1^ Bayesian Model Predicting Mean Response for Early Treatment Group (no imputed data)*
